# miRNAs as a Potential Biomarker in the COVID-19 Infection and Complications Course, Severity, and Outcome

**DOI:** 10.3390/diagnostics13061091

**Published:** 2023-03-14

**Authors:** Milena Jankovic, Dejan Nikolic, Ivana Novakovic, Bojana Petrovic, Milan Lackovic, Milena Santric-Milicevic

**Affiliations:** 1 Neurology Clinic, University Clinical Center of Serbia, 11000 Belgrade, Serbia; 2 Faculty of Medicine, University of Belgrade, 11000 Belgrade, Serbia; 3 Department of Physical Medicine and Rehabilitation, University Children’s Hospital, 11000 Belgrade, Serbia; 4 Clinic of Gynecology and Obstetrics, University Clinical Center of Serbia, 11000 Belgrade, Serbia; 5 Department of Obstetrics and Gynecology, University Hospital “Dragisa Misovic”, 11000 Belgrade, Serbia; 6 Institute of Social Medicine, Faculty of Medicine, University of Belgrade, 11000 Belgrade, Serbia; 7 Faculty of Medicine, School of Public Health and Health Management, University of Belgrade, 11000 Belgrade, Serbia

**Keywords:** COVID-19, miRNA, obesity, pregnancy, neurological diseases

## Abstract

During the last three years, since the emergence of the COVID-19 pandemic, a significant number of scientific publications have focused on resolving susceptibility to the infection, as well as the course of the disease and potential long-term complications. COVID-19 is widely considered as a multisystem disease and a variety of socioeconomic, medical, and genetic/epigenetic factors may contribute to the disease severity and outcome. Furthermore, the SARS-COV-2 infection may trigger pathological processes and accelerate underlying conditions to clinical entities. The development of specific and sensitive biomarkers that are easy to obtain will allow for patient stratification, prevention, prognosis, and more individualized treatments for COVID-19. miRNAs are proposed as promising biomarkers for different aspects of COVID-19 disease (susceptibility, severity, complication course, outcome, and therapeutic possibilities). This review summarizes the most relevant findings concerning miRNA involvement in COVID-19 pathology. Additionally, the role of miRNAs in wide range of complications due to accompanied and/or underlying health conditions is discussed. The importance of understanding the functional relationships between different conditions, such as pregnancy, obesity, or neurological diseases, with COVID-19 is also highlighted.

## 1. Introduction

COVID-19 is a viral infection caused by a single-stranded RNA virus [1]. In approximately 80% of cases, SARS-CoV-2 is associated with a silent or mild, flu like upper respiratory tract infection, and in the remaining 20% of cases, it may cause pneumonia, leading to a severe or critical form of infection that requires hospitalization and oxygen therapy [2]. Due to its severe pathogenicity and virulence, the virus has been the focus of the medical community since the beginning of the COVID-19 outbreak [3].

In addition to virus pathogenicity and virulence, so far identified several personal risk factors that determine the severity spectrum of COVID-19 infection among exposed people are older age, chronic comorbidities, gender, socioeconomic background, laboratory biomarkers, and ethnicity [4,5,6]. However, viral factors and host genetic differences have emerged as determinant for a patient’s susceptibility to develop severe form of SARS-CoV-2 infection [7]. Human genetic diversity is responsible for the modulated human immune response against SARS-CoV-2, explaining a wide spectrum of manifestations and heterogeneity of outcomes of SARS-CoV-2 infection [8]. At about the same time, associations identified between several genetic loci, rare genetic variants, and inborn errors of immunity with the disease [9,10,11] provide a window of opportunity for discovering new cellular-level interventions and drug therapies to remedy the patient’s gene dysregulation.

Numerous different therapeutic strategies, including antiviral, anticoagulant, anti-inflammatory, and antibody-based immunotherapeutic strategies, have been applied to decrease mortality associated with SARS-CoV-2 infection [12]. Recent scientific breakthroughs have presented an additional alternative-novel therapy, microRNAs (miRNAs), as a promising strategy for the deactivation of SARS-CoV-2 virus, decreasing risks to develop a severe form of the COVID-19 disease, for enabling normal biological processes or regulate genetic response/expression. miRNAs are natural, single-stranded, small RNA molecules that regulate gene expression by binding to target messenger RNAs (mRNAs), suppressing their translation or initiating their degradation, critical for defining cell identity during development and coordinating cellular activity throughout the cell’s lifetime [13]. These non-coding RNA molecules with around 21–23 nucleotides in length can regulate almost a third of a human genome and are involved in numerous pathways, including cell proliferation and death, fat metabolism, and stress resistance [14]. miRNAs play an important role in SARS-CoV-2–host interplay and viral replication by binding to the 3′-untranslated region (UTR) or 5′-UTR of viral RNA, or by affecting the synthesis of proteins associated with the SARS-CoV-2 life cycle, such as angiotensin-converting enzyme-2 (ACE2), transmembrane protease serine type-2 (TMPRSS2), Nsp12, and S proteins [15,16].

The role of previously described miRNAs in the pathogenesis of viral infections, particularly in coronavirus, is to participate in immune response and viral protein expression [17]. Furthermore, miRNA contributes to the fine-tuning of cell functions, which plays a significant role in innate and adaptive immune cell development [18]. Some of the miRNAs could be considered as key regulators of inflammation-related mediators [19]. Additionally, it was noted that host miRNAs can have role in the cytokine storm associated with a SARS-CoV-2 infection [18], as well as repression of SARS-CoV-2 genome expression [19].

It should be stated that the expression of miRNAs might be regulated by host cells leading to virus replication inhibition via complex pathways, including innate immunity pathway regulation or by the direct interaction with the viral genome [20]. Moreover, the expression of host genes could be mediated by the virus encoded miRNAs, thus unfavorably influencing the anti-viral host immune response [20]. Several genes can be regulated by a single miRNA and one gene can be targeted by multiple miRNAs [21]. We consider that three years since the emergence of SARS-CoV-2 is a relevant starting point to discuss possible evidence based prognostic and diagnostic parameters, such as circulating miRNA, for COVID-19 complications.

One aspect is to consider is SARS-CoV-2 infection as a direct cause of a multisystem disorder. Another approach is to assume that the infection can trigger some underlying conditions that have already started but not fully developed to clinical presentation, which is especially described in neurodegenerative diseases. Moreover, there is a possibility to consider the SARS-CoV-2 infection as part of the exposome and not to separate it from other environmental factors, socio-demographic components, and altered physiological and health conditions, such as pregnancy or obesity.

The aim of this review is to highlight the most relevant findings about miRNA as potential biomarkers for different courses and consequences of COVID-19 in the context of multimorbidity, with a focus on obesity, pregnancy, and neurological disorders.

## 2. Materials and Methods

In this study, we used an overview approach to survey the literature on miRNA in COVID-19 disease and summarize evidence to inform future research. The overview was conducted in five logical steps: (1) identification of relevant published articles, (2) study selection, (3) identification of the frequent topics, (4) description of main findings by topics, and (5) summary reporting. For the purpose of this review, 125 articles and reviews were analyzed. In the second step, the six most frequently studied topics were identified. These topics were: (i) miRNAs role in COVID-19 disease (ii) miRNAs as a modulator of cell protein ACE2; (iii) miRNAs against cellular mediator neuropilin-1 (NRP1); (iv) miRNAs role in obesity; (v) miRNAs role in pregnancy; (vi) miRNAs role in neurological diseases. In the third step, the main findings were described by topics outlining the miRNA modulation role in cell regulation (deregulation and upregulation) and gene expression (suppression and overexpression) in patients with COVID-19 disease and multimorbidity. In the final step, we provide a summary of the significance of the suppression and overexpression of genes, as well as their deregulation and upregulation, to highlight the relevance of discoveries for the treatment of COVID-19 patients and inform future drug and medicine research.

## 3. Results and Discussion

### 3.1. miRNAs Role in COVID-19 Disease

Saçar Demirci and Adan postulated that miRNAs could impact the virus in SARS-CoV-2 infection including the viral replication and translation as well as host expression modulation [22]. Furthermore, 67 different human miRNAs have been identified so far that target the spike protein of the SARS-CoV-2 virus [22,23]. In the study of Yang et al., the authors stated that certain miRNAs might inhibit the expression of proteins associated with the SARS-CoV-2 life cycle, including ACE2, TMPRSS2, Nsp12, and S proteins [16]. The role of ACE2 and TMPRSS2 in the SARS-CoV-2 infection was previously described [24]. It was noticed that the virus uses the ACE2 and TMPRSS2 to enter the cell and continue the infection, bearing in mind that not all cells and tissues express such enzymes [24].

Li et al. proposed in the study that 35 human miRNAs were significantly upregulated, by at least 1.5-fold, for miR-16-2-3p, miR-6501-5p, and miR-618, in COVID-19 patients versus the control group [25]. On the contrary, 38 human miRNAs were significantly downregulated, by 2.3-fold, for miR-627-5p and more than 1.3-fold, for miR-183-5p and miR-144-3p in COVID-19 patients versus the control group [25].

Donyavi et al. found in their comparative study of peripheral blood mononuclear cells that several inflammatory associated miRNAs, miR-29a-3p, miR146a-3p, and miR-155-5p, might be considered as potential biomarkers in distinguishing the acute phase of COVID-19 from healthy controls, miR-29a-3p, miR-146a-3p, and let-7b-3p as potential biomarkers in distinguishing post-acute phase of COVID-19 from healthy controls, while miR-29a-3p and miR-146a-3p might be considered as potential novel biomarkers in differentiating acute from the post-acute phase of COVID-19 disease [26].

Calderon-Dominguez et al. noticed that specific miRNAs (hsa-miR-32-5p, hsa-miR-98-3p, hsa-miR-423-3p, and hsa-miR-1246) were upregulated in COVID-19 critically ill patients compared to SARS-CoV-2 negative subjects. It is also worth noting that the levels of hsa-miR-32-5p and hsa-miR-1246 were elevated in critically ill COVID-19 compared to asymptomatic patients [27]. These findings might imply that certain miRNAs can be potential biomarkers for the severity of the COVID-19 disease. In addition to this, in another study, the following miRNAs were found to be significantly increased in severe COVID-19 patients versus the healthy group: miR-21 (fibrosis-associated miRNA), miR-155 (inflammatory miRNA), and miR-208a and miR-499 (heart-muscle specific miRNAs) [28]. In the discovery cohort, miR-126 was described to have protective effects on endothelia, and the damage was downregulated [28]. Furthermore, it was noticed that when adjusted for age and gender, miR-155, miR-146a, and miR-221 were shown to be significant in the identification of patients with severe COVID-19 disease, where miR-122-3p has the potential to target toll-like receptors (TLRs), tumor necrosis factor alpha (TNF-α), interleukin 6, interleukin 8, as well as transcription factors (NF-kB) that have essential roles in immune response [29]. Mc Donald et al. demonstrated, using the COVID-19 RNA sequencing patient data, that miR-2392 is the only upregulated miRNA [30]. Furthermore, miR-2392 increases inflammation, glycolysis, and hypoxia, and its concentration in blood and urine of COVID-19 patients is correlated with viral load and may serve as a prognostic biomarker [30].

The application of computational models in the prediction of miRNAs involvement in different subclinical and clinical health conditions may serve as a cost-effective approach in biomarker development. Numerous miRNA studies in COVID-19 disease applied deep machine learning models, recognized as crucial to identify the starting point and narrow down the number of possible research targets, according to internal loops or asymmetric bulges of pre-miRNAs [31,32]. On the contrary, without clinical samples and human cell lines, it is impossible to develop clinically relevant biomarkers [31].

### 3.2. miRNAs as a Modulator of Cell Protein ACE2

The inhibition of ACE2 can be achieved by enabling enzymatic function or by transcription suppression [33]. It should be underlined that ACE2 can be found in multiple organs, including the heart, kidneys, blood vessels, lungs, central nervous system, and digestive system [34]. Ahmed et al. stated that ACE2 is present in cardiomyocytes and that the presence of heart illness could lead to an increased level of *ACE2* expression [35]. The importance of ACE2 in coronavirus infection is in the fact that the receptor binding domain (RBD) found on S1 glycoprotein interacts with ACE2 receptors [33]. Such an interaction, particularly on endothelial cells, could lead to neurovascular damage, since aside from endothelial cells, *ACE2* is also expressed in different regions in the brain [36].

In the review of Singh et al., it was stated that certain miRNAs have a role in *ACE2* expression regulation. For example, miR-145 and miR-155 have been shown to upregulate *ACE2* gene, while miR-19b, miR-29, miR-132, miR-181, and miR-212 were shown to downregulate it [34]. However, specific miRNAs, such as miR-18 and miR-125b in kidneys, miR-4262 in lungs, and miR-146a in the heart, are described to be involved in *ACE2* expression regulation [34].

Previous studies have evaluated the role of miRNAs in SARS-CoV-2 cell entry. Has-miR-200c might have a role in reducing the risk of infection by inhibiting *ACE2* [23,37]. *ACE2* expression regulation by hsa-miR-125a, hsa-miR-141, and hsa-miR-200 family members was previously described in the study of Nersisyan et al. [38].

Moreover, Eyileten et al. noted that the most promising miRNAs that regulate ACE2-coagulation-related interactions networks are miR-27a, miR-16, let-7b, and miR-155 [39].

### 3.3. miRNAs as a Modulator of Cell Protein TMPRSS2

Previously, it was reported that TMPRSS2 plays an essential role in cleavage of ACE2 and S-protein, enabling viral entry through membrane fusion. Through the autocatalytic activity, TMPRSS2 becomes active and, in such form, cleaves the ACE2 receptor by interacting with the one [40]. It should also be stressed that *TMPRSS2* is considered as androgen-responsive gene [36]. The genetic variation of *TMPRSS2* may influence predisposition to viral infection as *TMPRSS2* rs8134378 polymorphism was found to increase expression in males and favor viral membrane fusion in viruses H1N1 and H7N9 [36].

Regarding potential miRNA candidates, it was suggested that miR-98 might regulate the expression of *TMPRSS2* in human endothelial cells [35,41]. Furthermore, additional miRNAs, including hsa-miR-32, hsa-miR-98, and hsa-miR-21, might be involved in TMPRSS2 synthesis suppression [35].

### 3.4. miRNAs against Cellular Mediator NRP1

In previous reports, aside from the ACE2, NRP1 might have a potential role in SARS-CoV-2 infection as a cellular mediator for SARS-CoV-2 entry into human cells [42]. Katopodis et al. noticed 69 miRNAs having a strong binding potential against NRP1 [42]. Furthermore, Naidoo et al. stated that several miRNAs might downregulate *NRP1* (miR-320(a-d), miR-124-3p, miR-214-3p, miR-206, miR-199a-5p, miR-24-3p, miR-130a-3p, and miR-141-3p), while it was pointed that miR-204 was overexpressed in COVID-19 disease [43]. Moreover, Mone et al. demonstrated that miR-24 targets 3`UTR of *NRP1* [44]. Additionally, they suggested that miR-24 might have a pathophysiological role in thromboembolic complications observed in COVID-19, while NRP1 might be involved in the cytokine storm in COVID-19 [44].

### 3.5. miRNAs Role in Obesity

Obesity is recognized as one of the most common comorbidities associated with COVID-19, with prevalence range from 30% to 60% among hospitalized patients [45]. Although numerous research has been carried out at the clinical, biochemical, and molecular levels, the connection between COVID-19 and obesity has not been fully clarified [46,47,48]. Given that infection susceptibility is viral lineage-dependent, it is interesting that the SARS-CoV-2 virus directly infects human adipocytes and changes their metabolism, particularly more visceral adipose tissue than subcutaneous [46,49]. It is known that adipose tissue is not only a fat depot in the body, but also an active factor with autocrine, paracrine, and endocrine roles. Obesity is considered a chronic low-grade inflammation, and adipose tissue produces numerous pro-inflammatory cytokines, such as IL6. Moreover, adipose tissue produces extracellular vesicles (EVs) that are released into the circulation and through which it communicates with other tissues [49,50,51]. The contents of these vesicles, among others, are micro RNA molecules of different classes. The micro RNA profile in obesity has been studied extensively even before the emergence of COVID-19, using animal models and human samples [52,53,54]. In the context of the SARS-CoV-2 infection, one of the lines of research was based on ACE2 as the main receptor for the entry of the SARS-CoV-2 virus into the cell and associated regulatory micro RNAs. Elemam et al. analyzed in COVID-19 patients the levels of soluble ACE2 (sACE2) and four micro RNAs (miR-421, miR-3909, miR-212-p, and miR-4677-3p) upstream regulators of ACE2 [48]. In this study, in relation to the body mass index (BMI), the subjects were divided into normal BMI, overweight, and obese, and elevated levels of sACE2 were found in all three groups, compared to healthy controls. Moreover, the values were higher in obese compared to overweight, which aligns with earlier findings outside of COVID-19. Three of four analyzed miRNAs (miR-421, miR-3909, and miR-4677-3p) were upregulated in all BMI groups, while miR-212-p was upregulated only in the group with normal BMI. In addition, levels of miR-212-p were strongly influenced by gender and disease severity also. This study stands out as the first designed in this way, and the authors stated as a limitation of the study that patients with severe COVID-19 were not included, as well as that possible usage of ACE inhibitors was not considered. On the other hand, Bellae Papannarao et al. identified that upregulation of miR-200c may increase the susceptibility to COVID-19 in obese persons [45]. They showed upregulation of miR-200c and miR-let-7b in patients with obesity. Concordantly, the ACE2 as a direct target for both miRNAs showed significant downregulation. However, studies showed that inhibition of ACE2 itself did not reduce the severity of COVID-19. On the contrary, a subsequent increase of angiotensin II may increase the severity of the disease. Since miRNAs are the earliest molecular regulators, the authors considered circulating miR-200c as a predictive biomarker for severe COVID-19. In her recent article, Roganovic presented the hypothesis that miR-146a deficiency contributes to the development of severe COVID-19 in obese people, as well as in people with hypertension or diabetes [55]. As part of the immune response to a virus, miR-146a is one of the first types of micro RNA to be induced, and it is known to act as a major downstream regulator of the TLR signaling pathway. It was also determined that the SARS-Cov-2 genome is a predicted target for that micro RNA. At multiple levels, the activity of miR-146a is to prevent an excessive immune response to the virus. Downregulation of miR-146a observed in obesity, as well as in hypertension and diabetes, may be one of the explanations for the association of these conditions with more severe forms and a worse outcome.

In her opinion paper, Mormile analyzed severe COVID-19 in young obese patients and highlighted the possible significant role of miR-126 downregulation [56]. It was established that, in obesity, visceral adipose tissue shows a functional abnormality, which is reflected, among others, in the reduced level of miR-126 in EVs. Downregulation of miR-126 may affect COVID-19 in several ways, and main is the increase of the risk for acute respiratory distress syndrome (ARDS). A recent discovery that delivery of miR-126 via EVs and exosomes is a novel mechanism of lung alveolar epithelium restoration. Both mature forms of the miR-126 gene products, miR-126-3p and miR-126-5p, play a role in alveolar epithelial renewal, while the level of miR-126-3p increases with age (age dependent). An additional method of downregulation is connected to the role of miR-126 as a suppressor of the production of several proinflammatory cytokines, including IL6. Therefore, miR-126 downregulation in COVID-19 enables cytokine storm. The third mechanism is via the influence of miR-126 on the occurrence of thrombosis, whereby circulating miR-126 reduces blood thrombogenicity. This is accomplished through post-transcriptional regulation of extracellular vesicle tissue factor (TF) expression. Consequently, the downregulation of miR-126 predisposes thrombotic events.

### 3.6. miRNAs Role in Pregnancy

Due to the physiological changes, especially the immunological and cardiovascular systems, which occur during pregnancy, pregnant women are at a higher risk for severe illness and a worse outcome during the SARS-CoV-2 infection. However, the death risk remains low [57,58]. Even though vertical transmission occurs rarely and can be symptomatic or asymptomatic, the risk of spreading the SARS-CoV-2 virus to the fetoplacental unit is considerable [59,60]. Pregnancy complications resulting from SARS-CoV-2 infection during pregnancy include miscarriage (2%), fetal growth restriction (10%), and premature labor (39%) [61].

Critical and severe COVID-19 disease correlates with preterm delivery in 75% and 9% of affected pregnancies, respectively, as well as with a higher risk of cesarean section [62,63].

A recent study based on Mendelian randomization provided evidence about shared genetic predisposition in women with a genetically determined SARS-CoV-2 infection for an 11.1% increased risk of hypertensive disorders during pregnancy [64].

Examining the impact of SARS-CoV-2 infection on gene expression, Gayen Nee’ Betal et al. found 374 up-regulated genes and 136 down-regulated genes in cord blood cells of patients infected by SARS-CoV-2 during pregnancy. Furthermore, they identified the modified canonical pathways related to embryonic and cellular development, cardiovascular and hematological diseases, and malignancy, as well as the altered toxicity functions that may contribute to cardiotoxicity, hepatotoxicity, and nephrotoxicity. They concluded that maternal systemic inflammatory response to SARS-CoV-2 infection in pregnancy could provoke epigenetic and various gene expression changes with potential consequences in childhood or adult life [65].

In a recent study, Saulle et al. revealed the upregulation of mi RNAs (miR-29a, miR-29c, miR-21, miR-98, miR-150, and miR-155), whose expression modulates viral infections, including SARS-CoV-2, in the placental biopsies of the SARS-CoV-2 infected pregnant women [66].

At the placental level, miR-21 expression may modulate infection by binding directly to a SARS-CoV-2 genome or interfering with SARS-CoV-2 replication by directly targeting its gene expression or interacting with multiple host transcripts, thus preventing a cytokine storm [66,67]. miR-98 may act as a transcription regulator, contributing to reduced infection risk and vertical transmission [41]. Furthermore, a study by Saullle et al. showed upregulation of some miRNAs involved in the innate and adaptive immune response, possibly by limiting tissue damage caused by inflammation, negative regulation of NK-dependent cytotoxicity, and maintaining the immune system hyperactivation, e.g., miR-146 and miR-150, in the placenta of COVID-19 pregnancies [66].

The expression of these miRNAs, e.g., miR-21, miR-29, miR-150, and miR-155, may lead to pregnancy-related complications, such as gestational diabetes, preeclampsia, intrauterine growth restriction, intrauterine hypoxia, and macrosomia [66,68,69].

Excessive expression of miRNA-155 has been observed during the host inflammatory response to viral replication [70]. Upregulation of miRNA-155 was described in the placentas of preeclamptic women [71]. Investigating the role of miRNA-155 in pregnancies with both preeclampsia and COVID-19 could lead to possible antagomirs treatment [72].

miRNA-126 which is decreased in preeclampsia was found to bind directly to the nucleocapsid of the SARS-CoV-2 [73,74]. Downregulation of miRNA-126 in preeclamptic pregnancies could lead to a higher risk for SARS-CoV-2 infection due to the loss of an anti-viral miRNA that targets SARS-CoV-2. Women with HIV-associated preeclampsia exhibit a decrease in miRNA-126, suggesting that women with both conditions could have a higher risk for SARS-CoV-2 infection and severe disease [72].

IgG antibodies to the SARS-CoV-2 virus cross the placenta, which is why immunization against this virus is recommended for women in the reproductive period [75]. Comparing vaccinated and unvaccinated gravid women, Lin et al. detected different expressions of the seven miRNAs [76]. It was stated that miRNA-1972, miRNA-191-5p, and miRNA-423-5p were overexpressed in the vaccinated, and miRNA-16-5p, miRNA-486-5p, miRNA-21-5p, and miRNA-451a were overexpressed in unvaccinated pregnant women [76]. New technologies produced vaccines that contain messenger RNA (mRNA) of the spike protein from the surface of the SARSC-CoV-2 virus. As these vaccines do not contain the virus, and the host’s cells decompose the mRNA in several days, it is considered that mRNA vaccines are the most suitable for use during pregnancy [76].

### 3.7. miRNAs Role in Neurological Diseases

Several years now since the emergence of the COVID-19 pandemic, numerous studies have reported that patients with neurological diseases are at higher risk to develop more severe symptoms after SARS-CoV-2 infection. Various complications and higher mortality rates are confirmed in patients with progressive autoimmune disorders, such as multiple sclerosis (MS), but also in neurodegenerative disorders, such as Alzheimer’s disease (AD) and Parkinson’s disease (PD) [77,78,79]. Similar trends were reported in very rare neurodegenerative disorders, such as amyotrophic lateral sclerosis/frontotemporal dementia (ALS/FTD) and prion disease, although with less data [80,81].

Viral, bacterial, or other infections of the nervous system showed the ability to trigger or accelerate neuroinflammation and neurodegeneration, subsequently leading to various clinically relevant manifestations [82,83,84]. Most of those disorders are age-dependent, suggesting that the ageing brain is more vulnerable to inflammatory degeneration provoked by a cytokine storm [85,86,87]. Additionally, severe COVID-19 has been observed in older people, who are already at higher risk of developing neurological disorders, even without SARS-CoV-2 infection [88]. The possibility of the preclinical phase of underlying molecular and physiological neurodegenerative processes triggered by a viral infection should not be excluded [89].

**Figure 1:**
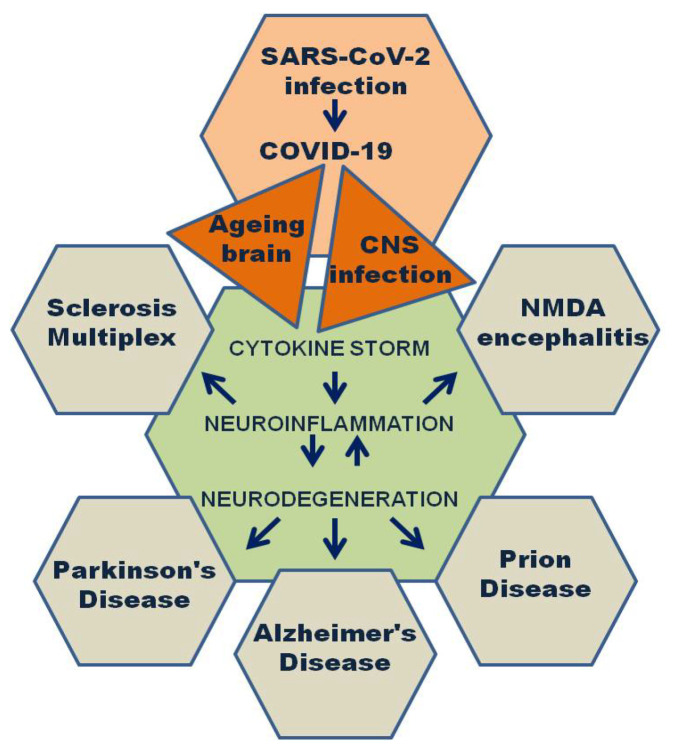
Schematic representation of COVID-19 correlation with neurological diseases, based on study findings.

Another aspect of the association between SARS-CoV-2 infection and neurological diseases is presented through a broad spectrum of neurological manifestations during and after the acute COVID-19 phase. As COVID-19 is considered a multisystem disorder, it may affect different organs for a prolonged period as, including the central nervous system (CNS) [90]. Acute, progressive, and chronic CNS consequences of the SARS-CoV-2 infection are developed through the synergic action of different mechanisms of the viral life circle. As described in previous sections, the ACE2 receptor is abundant in the CNS, particularly in the medulla oblongata and pons in the brainstem and choroid plexus and visual areas of the occipital lobe [91,92]. Additionally, the S1 spike protein has numerous prion-like characteristics, especially the propagation of atypical and progressive aggregation of misfolded proteins [89,93]. This mechanism is supported through numerous case reports of patients with COVID-19 and prion disease [86,94,95,96,97]. Another frequent neurological consequence is anti-NMDA encephalitis, reported in pediatric and adult COVID-19 patients [98], with possible overlapping prion disease (Figure 1) [99].

Early clinical presentation of the vast majority of neurodegenerative disorders is overlapping, and first symptoms are detectable when the pathological processes on a molecular level are far more advanced, and thus irreversible and unstoppable. Great interest in developing efficient biomarkers for COVID-19 overlaps with the importance of biomarkers in neurodegeneration, and miRNAs are widely proposed.

As described above, serum levels of several miRNAs have been shown to be altered in COVID-19 patients and proposed as potential biomarkers for distinguishing acute from post-acute phase of the disease [26]. Interestingly, several miRNAs show the same expression alterations in neurological disorders, providing the opportunity to be considered biomarkers for neurological complications of COVID-19.

miR-155 is elevated in COVID-19 patients and correlates with disease severity [100]. Additionally, in several neurological disorders, such as MS, AD, and Down’s syndrome dementia. This proinflammatory miRNA overexpression accelerates inflammation in the CNS trough targeting various anti-inflammatory regulators, increasing the permeability of the blood–brain barrier and T lymphocyte activation, leading to beta-amyloid plaque formation [101,102,103,104]. Let-7b is another miRNA that is overexpressed in COVID-19 patients and in neurodegenerative disorders, such as AD and PD, and a possible common mechanism is apoptosis promotion [105,106]. This miRNA has been downregulated in anti-NMDA encephalitis and proposed as a biomarker for this condition [107]. Several other miRNAs from the same family (let-7a, let-7d, and let-7f) have been reported to be significantly less expressed in anti-NMDA encephalitis patients, and the majority of them were also associated with other neurological disorders [107]. Comparing over 50 miRNAs differentially expressed in COVID-19 with miRNAs directly or indirectly associated with anti-NMDA encephalitis revealed seven common miRNAs: miR-107, miR-29b, let-7a, let-7f, miR-26b, miR-21, and miR-155 [108].

On the contrary, serum levels of several miRNAs are found to be decreased during COVID-19 as well as in neurological diseases. Downregulated expression of miR-21, which targets proinflammatory genes, has a proinflammatory effect in COVID-19 patients and increases the severity of the disease, but also affects neuroinflammation and may be a valuable treatment target [109]. miR-31 targets several PD, AD, and multiple system atrophy (MSA)-associated genes, and decreased expression may lead to neurodegeneration [110]. On the other hand, expression studies of miR-31 in COVID-19 patients show conflicting results [111,112]. Another COVID-19 associated microRNA, silencing genes associated with neurodegenerative disorders, is miR-16 [113]. The reverse correlation of mir-16 serum level and COVID-19 severity has been reported, proposing its potential to become a prognostic biomarker [114]. Additionally, this molecule is recognized as a potential therapeutic agent. It is intriguing that miR-16, together with miR-146, was previously reported to be involved in the early stages of prion disease, and, on the other hand, as a potent biomarker for types of pneumonia [115,116]. miR-146 is an abundant miRNA in the brain, which is upregulated in models of neurodegenerative disorders and upon infection [117,118,119,120]. This miRNA is proposed as a neuroprotective agent, according to the expression of a strong negative effect on neuroinflammation. Overexpressed miR-146 reduces cognitive decline and beta-amyloid plaque formation and promotes the clearance of misfolded proteins [121]. Moreover, it is considered a clinical biomarker for neurodegeneration as levels of circulating miR-146 are significantly reduced in patients with AD [118,119]. The expression of miR-146 is altered in COVID-19 patients [26] and may indicate a worse prognosis in patients with other inflammatory diseases, such as obesity, diabetes, or hypertension [47,55].

A correlation with chronic fatigue has emerged as the main focus shifted from acute SARS-CoV-2 infection to chronic consequences [122]. Chronic fatigue syndrome (CFS) is a multisystem condition with heterozygous presentation and often overlaps with other neurological disorders and possibly shares similar pathophysiological mechanisms [123]. Numerous miRNAs differentially expressed in CSF are also involved in the regulation of immune response, and miR-558, miR-146a, miR-150, miR-124, and miR-143 are directly associated with inflammatory-related genes [124,125]. The similarity between CSF and long-COVID-19 may accelerate further research in identifying previously unknown common pathological pathways.

A summary of the review findings regarding the different role of miRNAs in COVID-19 disease among patients with obesity, neurological disorders, and among pregnant women is presented in Table 1.

**Figure 2:**
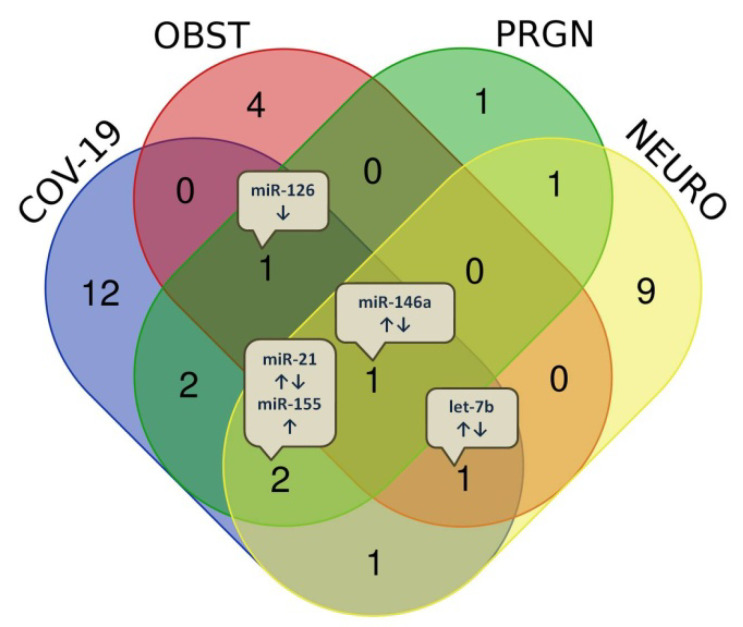
Numbers of overlapping of representative miRNAs in several different physiologically altered conditions, associated with COVID-19. The alternatively expressed miRNAs are presented in text bubbles. COV-19—covid-19; OBST—obesity; PRGN—pregnancy; NEURO—neurological diseases.

Although pregnancy, obesity, and neurological diseases may be considered quite diverse physiological and metabolic conditions, it is possible that their molecular pathways intersect and that the same miRNAs are involved (Table 2; Figure 2). miR-146a is the only alternatively expressed microRNA in all observed conditions (Figure 2).

The same miRNAs may be regulators of numerous biological processes, including immune response, inflammation, and subsequently informative biomarkers, and could be considered as the targets in therapy for several conditions.

## 4. Conclusions

This review outlined that miRNA regulation in SARS-CoV-2 infection is functionally related with pregnancy, obesity, and neurological disorders. Hence, a profile of circulating miRNA in the different stages of COVID-19 disease and patient stratification according to their miRNA status may provide important clinical insights, indicating the direction for further interventions.

The great importance of measuring molecular changes, not only during the acute phase of the disease, but also after the contagious stage, is pointed out. Recent studies are focused on estimating the increased risk of long-term health problems after the acute phase of the disease and developing informative biomarkers for targeted prevention.

We summarized studies that recognized miRNAs as adequate biomarkers that will allow individualized follow-up of the disease progression and concluded that early recognition of patients at high risk for COVID-19 complications and developing chronic and progressive conditions will provide the opportunity for better treatments in possibly reversible phases of the disease.

Moreover, the review considers the progress made and obstacles remaining in the clinical application of miRNA biomarkers in combination with other metabolic patterns. The main direction in future miRNA studies should focus on how miRNAs may be informative as part of the set of different biomarkers and not to search for the “Holy Grail” biomarkers. Additionally, the development of technologies to provide standardized and simple laboratory procedures will be the steppingstone for clinical application.

## Figures and Tables

**Table 1:** Selected miRNAs differentially expressed in patients with COVID-19 and patients with COVID-19 and conditions: obesity, pregnancy or neurological disorders. ↑—upregulated; ↓—downregulated.

COVID-19	COVID-19 and Obesity	COVID-19 and Pregnancy	COVID-19 and Neurological Disorders
miR-16 ↑ miR-6501 ↑ miR-618 ↑ miR-627 ↑ miR-183 ↓ miR-144 ↓ miR-29a ↑ miR-146a ↑↓ miR-155 ↑ let-7b ↑ miR-98 ↑ miR-423 ↑ miR-1246 ↑ miR-32 ↑ miR-21 ↑ miR-208a ↑ miR-499 ↑ miR-126 ↓ miR-221 ↑ miR-2392 ↑	miR-421 ↑ miR-3909 ↑ miR-4677 ↑ miR-200c ↑ let-7b ↑ miR-146a ↓ miR-126 ↓	miR-29a ↑ miR-29c ↑ miR-21 ↑ miR-98 ↑ miR-150 ↑ miR-155 ↑ miR-146a ↑ miR-126 ↓	miR-155 ↑ let-7b ↑↓ miR-107 ↑ miR-29b ↑ let-7a ↓ let-7f ↓ miR-26b ↑ miR-21 ↓ miR-31 ↓ miR-16 ↑ miR-146a ↑↓ miR-558 ↓ miR-150 ↓ miR-124 ↓ miR-143 ↓

**Table 2:** A list of same miRNAs involved in COVID-19 and patients with COVID-19 and conditions. ↑—upregulated; ↓—downregulated.

Diseases Overlap	miRNAs, n	miRNAs, Name
COVID-19 Obesity Pregnancy Neurol. diseases	1	miR-146a ↑↓
COVID-19 Obesity Pregnancy	1	miR-126 ↓
COVID-19 Obesity Neurol. diseases	1	let-7b ↑↓
COVID-19 Pregnancy Neurol. diseases	2	miR-21 ↑↓ miR-155 ↑
COVID-19 Pregnancy	2	miR-29a ↑ miR-98 ↑
COVID-19 Neurol. diseases	1	miR-16 ↑
Pregnancy Neurol. diseases	1	miR-150 ↑
